# A review of molecular beam epitaxy of ferroelectric BaTiO_3_ films on Si, Ge and GaAs substrates and their applications

**DOI:** 10.1088/1468-6996/16/3/036005

**Published:** 2015-06-30

**Authors:** Lucie Mazet, Sang Mo Yang, Sergei V Kalinin, Sylvie Schamm-Chardon, Catherine Dubourdieu

**Affiliations:** 1 Institut des Nanotechnologies de Lyon, CNRS, Ecole Centrale de Lyon, Université de Lyon, 69134 Ecully, France; 2 Center for Nanophase Materials Sciences, Oak Ridge National Laboratory, Oak Ridge, TN 37831, USA; 3 CEMES-CNRS, Université de Toulouse, 29 rue Jeanne Marvig, F-31055 Toulouse, France

**Keywords:** molecular beam epitaxy, ferroelectric, semiconductor

## Abstract

SrTiO_3_ epitaxial growth by molecular beam epitaxy (MBE) on silicon has opened up the route to the monolithic integration of various complex oxides on the complementary metal-oxide–semiconductor silicon platform. Among functional oxides, ferroelectric perovskite oxides offer promising perspectives to improve or add functionalities on-chip. We review the growth by MBE of the ferroelectric compound BaTiO_3_ on silicon (Si), germanium (Ge) and gallium arsenide (GaAs) and we discuss the film properties in terms of crystalline structure, microstructure and ferroelectricity. Finally, we review the last developments in two areas of interest for the applications of BaTiO_3_ films on silicon, namely integrated photonics, which benefits from the large Pockels effect of BaTiO_3_, and low power logic devices, which may benefit from the negative capacitance of the ferroelectric.

## 1. Introduction

Complex oxides exhibit a wide range of electrical, magnetic, optical and mechanical properties, which may even be coupled. This extraordinary wealth of physical properties offers a huge potential for developing new functionalities in devices that can address societal needs related to health, energy efficiency or information and communication technologies. Ferroelectrics are particularly attractive for their applications in nanoelectronics, communication devices, electro–mechanical systems or sensors. However, in order to exploit their properties, complex oxide integration should be performed in a seamless manner on a semiconductor platform such as silicon or III/V substrates in order to be compatible with the mainstream nanoelectronic industry.

A great variety of complex oxides crystallize in a perovskite-type structure [1]. Tremendous progress has been achieved in the growth of oxides on oxide substrates (such as SrTiO_3_, LaAlO_3_, scandates, Al_2_O_3_, MgO…) in the past 15 years. Unit cell control (∼4 Å) of the growth can now be achieved. New phenomena arising from interfaces have emerged [2–5]. Progress in characterization techniques and modeling (density functional theory (DFT)) have allowed the study of physico-chemistry and mechanisms involved at the nanoscale and have resulted in a better understanding of the effect of size, strain and boundaries conditions on the properties of complex oxides.

Integrating a perovskite oxide epitaxially on silicon is much more difficult and is still in its infancy, particularly regarding practical devices. One major difficulty for the epitaxy lies in the necessity to avoid the formation of an amorphous SiO_2_ interfacial layer in the first stages of the growth. The first direct epitaxy of a perovskite (SrTiO_3_) on silicon was realized by molecular beam epitaxy (MBE) in 1998 [6]. MBE provides unique advantages to precisely construct, almost atom by atom, the oxide/semiconductor interface. Although this breakthrough achievement showed promise in integrating—in a monolithic way—oxides on a semiconductor platform, only a few successes have been reported in the past 15 years [7].

In this contribution to the focus issue on ‘Properties and Applications of Perovskites’, we discuss the monolithic integration of complex oxides on semiconductors by MBE. We will illustrate the particular case of the ferroelectric BaTiO_3_ compound. We review the work in the literature as well as our own work. The crystalline structure and the ferroelectric properties of BaTiO_3_ heterostructures on Si, Ge and GaAs are presented. Finally, an overview of perspectives and recent progress in ferroelectric oxide integration on semiconductors for low power logic devices and integrated photonics is provided.

## 2. MBE of complex oxides: a brief introduction to the technique

MBE was originally developed to epitaxially grow III–V compound semiconductors on a crystalline substrate [8, 9]. It allows a control almost atom by atom of the growth in ultrahigh vacuum conditions. Progress in the MBE of oxides as well as in other deposition techniques took off in the late 1980s, after the discovery of the high-*T*_c_ superconductor YBa_2_Cu_3_O_7-*δ*_. This implied the design of dedicated metal-oxide growth MBE chambers. MBE of crystalline oxides on silicon and on other semiconductor substrates has been developed in the late 1990s when the first epitaxy of SrTiO_3_ on Si was demonstrated [6]. This research benefited from the huge amount of efforts triggered by the microelectronic industry on the search for high-permittivity (high *κ*) oxides as a replacement to SiO_2_ (or SiON) gate oxide in complementary metal-oxide–semiconductor (CMOS) field-effect transistors.

In the MBE of complex metal oxides, Knudsen effusion cells commonly used to evaporate the metals (Ba, Sr, Ti…) are focused onto a heated substrate under ultrahigh vacuum conditions (typically 10^−10^–10^−9^ Torr). In the case of refractory elements, such as Ti, various means of evaporation have been reported: effusion cells, e-beam gun evaporation, Ti-Ball sublimation source [10] and metal-organic vapor source [11, 12]. Metal organic sources used in metalorganic chemical vapor deposition [13] such as titanium tetra isopropoxide Ti(OCH_3_H_7_)_4_ have been proposed in order to increase by several orders of magnitude the vapor pressure of Ti as compared to solid source and to have a beam flux that is relatively unaffected by the presence of oxygen in the chamber [12]. A wide window of growth parameters with self-regulating stoichiometry has been reported for SrTiO_3_ films grown using such a hybrid approach combining a conventional Sr effusion cell with a metalorganic precursor source for Ti [12].

For a given compound, deposition occurs by alternating the individual flux by means of shutters or by co-directing all fluxes simultaneously towards the substrate. Either molecular oxygen or atomic oxygen generated by a plasma source is typically used to provide the necessary oxygen to form the oxide. The oxygen pressure ranges typically from the 10^−8^ to 10^−5^ Torr. The background oxygen pressure plays a major role on the final stoichiometry, crystalline orientation and roughness of the films, as we will show later.

The reactivity of metal elements (in the chamber and in the sources) with the ambient oxygen (typically in the high 10^−8^–10^−5^ Torr range) makes the control of the beam fluxes, and therefore composition, difficult. This is actually a major issue in the MBE of multicomponent oxides, which can usually accommodate a large range of non-stoichiometric composition. Indeed the physical properties of complex oxides are strongly dependent on the cationic and oxygen stoichiometry. Moreover, composition deviation may lead to the formation of spurious phases. A quartz crystal microbalance may be used to measure the flux of each atomic beam at the position of the substrate but it does not allow simultaneous monitoring of each flux (it is not element specific) and cannot be employed for *in situ* control of the composition. *In situ* monitoring techniques have been proposed for composition control of multi-element oxides [14]. Among them, reflection high-energy electron diffraction (RHEED) is commonly used and has proven to be an effective *in situ* and real-time diagnostic tool. A review article is proposed in [15] for the use of RHEED during complex oxide growth. It allows one to follow in real time the crystallinity of the deposited film and to adjust in real time the composition by tuning the impinging fluxes when additional spots originating from spurious phases are observed on the RHEED pattern.

Haeni *et al* [16] have proposed to use RHEED oscillations, both their shape and intensity, to control in real time the composition of multicomponent oxides such as SrTiO_3_. They reported a control to within 1% of Sr:Ti ratio by monitoring the shuttered RHEED oscillations as the substrate surface is sequentially exposed to the Sr or Ti fluxes. This precise control of monolayer (ML) doses of Sr and Ti has been used to successfully grow the first five members of the Sr_*n*+1_Ti_*n*_O_3*n*+1_ Ruddlesden–Popper phases [17, 18].

Various complex oxides have been grown by MBE. As mentioned, the development of oxide MBE started after the discovery of the high *T*_c_ cuprate superconducting compounds [19–28]. Since then, a variety of multiple-cation oxides have been epitaxially deposited by MBE on oxide substrates: SrTiO_3_, Ruddlesden–Popper phases, Bi_4_Ti_3_O_12_, Ba(Sr)TiO_3_, SrVO_3_, GdTiO_3_, BiFeO_3_, LaAlO_3_, PbTiO_3_, LaCrO_3_, SrCrO_3−*δ*_, La_1−*x*_Sr_*x*_FeO_3_, LaTiO_3.5_, La_2_Zr_2_O_7_, LaNiO_3_, La_2_NiO_4_, LaSrAlO_4_, and superlattices e.g. BaTiO_3_/SrTiO_3_ or PbTiO_3_/ SrTiO_3_ to name only a few compounds and groups [29–54]. On Si substrates, epitaxial SrTiO_3_ films are used as templates to grow a variety of complex oxides. BaTiO_3_ has been the most studied one by MBE. Apart from this compound, relatively few complex oxides (perovskite, spinel, pyrochlore phases…) have been grown by MBE on Si [55–62]. In many cases, the epitaxial growth on the template layers is completed using other deposition techniques such as pulsed laser deposition, sputtering, chemical vapor deposition or atomic layer deposition, as reported in [64–69] for BaTiO_3_.

A review of crystalline oxides on silicon is provided in [7]. The paper by Baek and Eom [63] gives a recent review of the epitaxial integration on silicon using SrTiO_3_ templates of the multiferroic BiFeO_3_, of the relaxor Pb(Mg_1/3_Nb_2/3_)O_3_–PbTiO_3_ (PMN-PT) and of LaAlO_3_/SrTiO_3_ heterostructures for 2D electron gas creation at their interface.

In the following, we focus on the MBE of the ferroelectric compound BaTiO_3_ on silicon, germanium and gallium arsenide and on the related crystalline and ferroelectric properties.

## 3. MBE of BaTiO_3_ on semiconductors: growth and crystalline structure

BaTiO_3_ is a prototypical ferroelectric perovskite oxide, with a Curie temperature of 120 °C. The ferroelectric tetragonal structure has lattice parameters of *a* = 3.994 Å and *c* = 4.0335 Å with space group P4mm (ICDD #83–1880) and the cubic paraelectric one has a lattice parameter of 4.006 Å with space group Pm-3m (ICDD #79–2263). The polarization is aligned along the *c*-axis of the tetragonal lattice. The tetragonality ratio *c*/*a* is 1.01, which is smaller than in Pb-based ferroelectrics such as PbTiO_3_ (*c*/*a* = 1.04). BaTiO_3_ is an attractive ferroelectric for nanoelectronic, energy harvesting and photonic applications as will be discussed later in this article. It is a lead-free compound, which is an advantage regarding European regulation and industrial clean room compatibility.

While most MBE depositions of BaTiO_3_ on a semiconductor have been carried out on silicon, there is a growing interest in Ge and GaAs.

Silicon is the major semiconductor industry substrate. Current CMOS technologies are based on silicon wafers with size up to 300 mm and technologies on 450 mm wafer size are under development. Germanium (also a group IV semiconductor) is of high interest for field-effect transistors with p-type channel (p-FETs) due to the higher mobility of holes as compared to Si. Biaxially strained SiGe channels on Si have also recently attracted much attention for p-FETs. Both Si and Ge have a diamond structure with lattice parameter of 5.431 Å and 5.658 Å respectively. They form a solid solution Si_1−*x*_Ge_*x*_ in the entire composition range (0 ≤ *x* ≤ 1).

The III–V gallium arsenide semiconductor has higher electron mobility than Si, which makes it attractive for n-FETs. It is today extensively studied as a channel for advanced CMOS technologies. GaAs has also a wider band gap than Si making it highly resistive if undoped. It is also more resistive to heat and radiation damage. It is suited for many applications such as high frequency devices in communications or such as microwave and millimeter wave integrated circuits. Another advantage of GaAs is its direct band gap, which is of interest for optical applications. GaAs has a zinc blende structure with a lattice parameter of 5.653 Å.

BaTiO_3_ deposition is mostly performed using an oxide template since the direct epitaxy on semiconductors would result in a high defect density or in a non-appropriate film orientation. We thus describe the direct growth of SrTiO_3_ epitaxial films on semiconductors when relevant and their use for the epitaxial growth of BaTiO_3_.

In the following, BaTiO_3_ crystalline domains with respectively the *c*-axis or the *a*-axis of the tetragonal cell being out-of-plane relatively to the substrate (001) plane are denoted respectively *c*-domains and *a*-domains.

### 3.1. MBE of BaTiO_3_ on silicon

The lattice mismatch (*a*_Si_–*a*_BTO_)/*a*_BTO_, between BaTiO_3_ and Si(001) is about 4%, which is quite large and tends to favor *a*-axis growth when BaTiO_3_ is directly grown on Si [70]. Moreover, the large mismatch of the thermal expansion coefficients between Si (*α* = 2.6 × 10^−6^ K^−1^) and BaTiO_3_ (*α* = 9 × 10^−6^ K^−1^) leads to an in-plane biaxial tensile strain exerted on BaTiO_3_ upon cooling, which favors *a*-axis growth. In order to obtain *c*-axis oriented BaTiO_3_ films on silicon, a buffer layer that exerts a biaxial compressive in-plane strain should be used to overcome the biaxial tensile in-plane strain during cooling to room temperature [71]. SrTiO_3_ has been widely used for such a purpose.

#### 3.1.1. SrTiO_3_ epitaxial templates on Si

The pioneering work of McKee and co-workers [6] opened up the route to the epitaxial growth of perovskite-type compounds on silicon and more generally to any oxide that could be epitaxially grown on bulk SrTiO_3_ substrates.

SrTiO_3_ is probably the most investigated epitaxial oxide on silicon [7, 72–100]. Many studies have been directed towards understanding the crystalline and electronic structure of the film and of its interface with Si (or SiO_2_).

The epitaxial growth is realized by passivating the clean Si(001) 2 × 1 reconstructed surface by ½ of a ML of Sr. The Sr atoms are positioned between the Si dimers and prevent the surface from oxidizing. The native SiO_2_ can be *in situ* thermally removed at high temperature; the clean Si (001) surface is then passivated by dosing the Sr metal to ½ ML. SiO_2_ can also be removed using a strontium-assisted deoxidation process in which Sr acts as a catalyst to desorb the native oxide [74]; in this case, once SiO_2_ is fully desorbed, more Sr is deposited until a 2 × 1 reconstructed surface appears on the RHEED, indicating the passivation of the Si(001) surface with ½ ML Sr coverage. The subsequent growth of SrTiO_3_ can be performed in different ways. The first few MLs have to be grown at low temperature in order to avoid the oxidation of the interface.

Commensurate SrTiO_3_ thin films may be grown on Si(001) using a sequential process named ‘kinetically controlled sequential deposition process’ [75, 82]. The growth proceeds by alternating a low temperature deposition (∼200–300 °C) of 1–3 ML of a mainly amorphous Sr–Ti–O compound under an oxygen pressure of typically 10^−8^–1.5 × 10^−7^ Torr, followed by an annealing step at higher temperature (580–700 °C) in ultrahigh vacuum conditions (<5 × 10^−9^ Torr) to crystallize the SrTiO_3_ phase. When grown in such conditions, there is no interfacial SiO_2_ oxide formed and SrTiO_3_ films have in plane lattice parameter commensurate to the Si 1 × 1 lattice. Relaxation occurs for ∼5 ML. Ferroelectricity in such ultrathin compressively strained films has been reported [89].

The SrTiO_3_ deposition may also be performed by growing at a higher temperature after the first few MLs of SrTiO_3_ have been grown, with a low temperature growth/high temperature post-anneal. In this case, the higher growth temperature (>450 °C) under oxygen results in an amorphous SiO_2_ interfacial layer due to oxygen diffusion through the film down to the interface with silicon. Since this amorphous layer occurs after the direct epitaxy of SrTiO_3_ on Si, it does not disrupt the epitaxy of the SrTiO_3_ film and subsequent oxide growth. The epitaxial relationship between SrTiO_3_ and Si, due to the lattice mismatch, is: [100]_SrTiO3_//[110]_Si_ and (001)_SrTiO3_//(001)_Si_.

**Figure 1.**
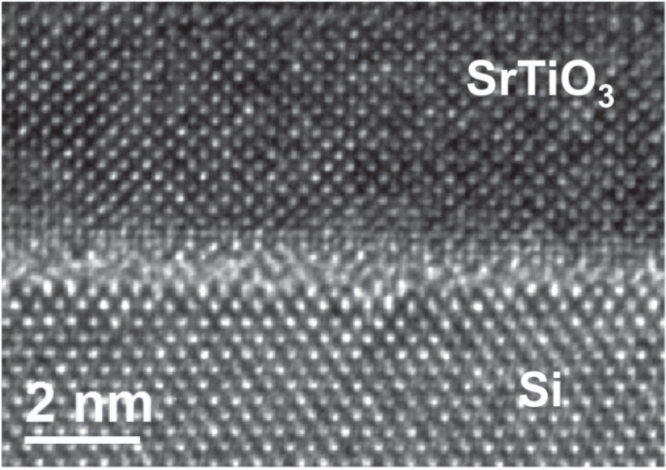
High-resolution TEM image of a SrTiO_3_ thin film deposited on Si (001) substrate by MBE.

In figure 1, we show a high resolution transmission electron microscopy (TEM) image of a SrTiO_3_ film deposited on Si substrate at a temperature of 400 °C under an oxygen partial pressure of P(O_2_) = 5 × 10^−8^ Torr followed by a crystallization step at 460 °C for 20 min under ultrahigh vacuum. We used a rapid cooling down procedure followed by a plasma anneal at 200 °C for 40 min in order to minimize the SiO_2_ regrowth while providing oxygen to the SrTiO_3_ lattice.

**Figure 2.**
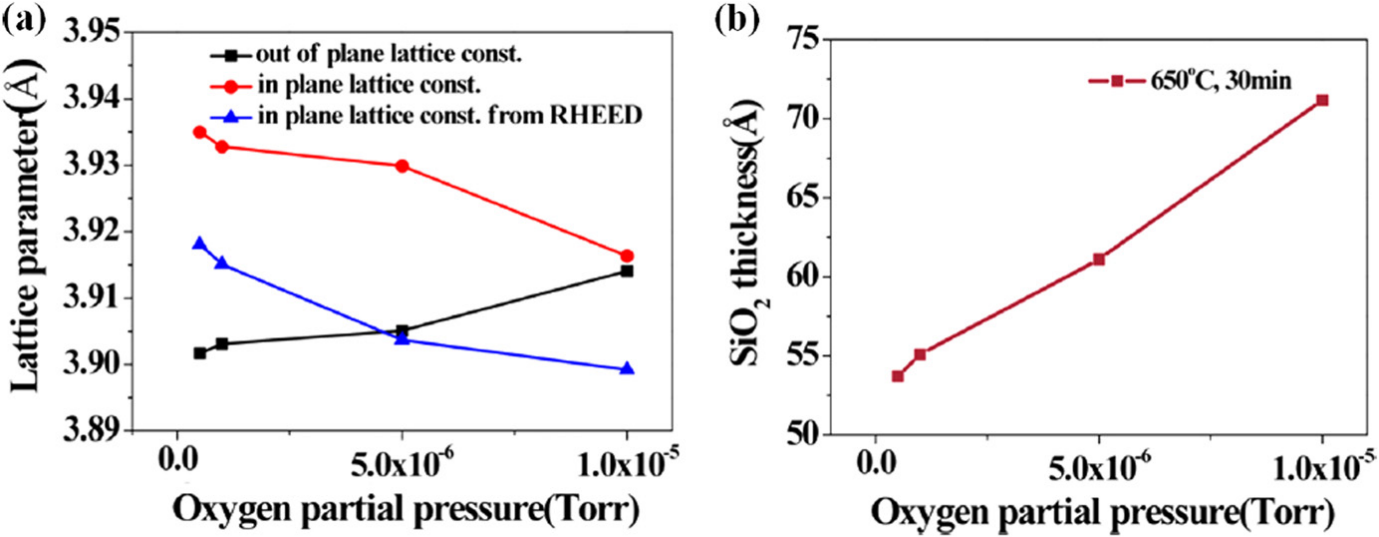
(a) In-plane and out-of-plane lattice constants and (b) SiO_2_ thickness as a function of oxygen partial pressure. All films were annealed at 650 °C for 30 min in different oxygen environments. From figure 5 in [96]. Reprinted with permission from M Choi *et al* 2012 *J. Appl. Phys.* **111** 064112. Copyright 2012, AIP Publishing LLC.

Choi *et al* showed that the SiO_2_ interfacial layer thickness increased during post-deposition annealing as P(O_2_) and/or annealing time were increased (annealing at 650 °C under P(O_2_) from 2 × 10^−7^ to 1 × 10^−5^ Torr) [96] and that it can be used to tune the strain relaxation of the SrTiO_3_ layer. Before annealing, the SrTiO_3_ layers are expanded in-plane due to the bi-axial tensile strain exerted by Si during cooling down. As the oxygen partial pressure is increased during the post-deposition anneal, the SrTiO_3_ lattice parameters evolve towards those of a cubic structure, which is concurrent to the SiO_2_ interlayer thickness increase (figure 2). Strain can be tuned in the SrTiO_3_ films within half a per cent, which can be useful to adapt the lattice constants to the oxide to be grown on top [96].

Thick SrTiO_3_ films (100 nm) grown by MBE and annealed at high temperature (900 °C) exhibit a full width at half maximum (FWHM) of the 002 rocking curve much narrower than the one of a bulk single crystalline substrate (the quality of which may, however, vary considerably depending on the quality of the original crystal) [90]. A TiO_2_-terminated surface similar to the one typically prepared on bulk single crystalline SrTiO_3_ substrates could be obtained by buffered HF etching of the annealed films [90]. This procedure requires, however, thick films since interfacial reactions occur at high temperatures. On thinner 1–4 nm SrTiO_3_ templates on Si, such a post-deposition annealing at 900 °C is not feasible. The surface may be TiO_2_ terminated by switching off the Sr beam and properly dosing the Ti flux.

#### 3.1.2. MBE of BaTiO_3_ on SrTiO_3_-buffered Si

In their pioneering work [71], the group of Schlom used the solid solution Ba_1−*x*_Sr_*x*_TiO_3_ as a buffer and could obtain fully *c*-axis oriented BaTiO_3_ films, while previous attempts to grow BaTiO_3_ on silicon had lead to *a*-axis films. Both the Ba_0.7_Sr_0.3_TiO_3_ buffer and the BaTiO_3_ films were grown by MBE. A thickness of about 10 nm was estimated for the buffer to be relaxed, which was the condition to obtain *c*-axis BaTiO_3_ growth. In these conditions, a 10 nm BaTiO_3_ film was commensurate with the buffer (30 nm) and had an in-plane lattice parameter of 3.9996 ± 0.0005 Å, indicating that the film was predominantly *c*-axis oriented. This result was corroborated by optical second harmonic generation measurements. Shortly after, the group of Wessels [101] demonstrated the growth of *c*-axis BaTiO_3_ using a 5 ML SrTiO_3_ template (∼2 nm). By varying the film thickness, they observed that the BaTiO_3_ growth started as pseudomorphic and that strain relaxation occurred at a critical value of 10 ML (∼4 nm). The out-of-plane lattice parameter was found to be fully relaxed at about 30–40 nm. They observe a mixed *a-* and *c-*oriented domain structure and the values extracted from a *θ*/2*θ* x-ray diffraction scan were *a* = 4.01 Å and *c* = 4.05 Å. Niu *et al* [102] reported the growth of a 40 nm BaTiO_3_ fully *c*-axis film on SrTiO_3_-buffered (5 nm thick) Si substrate, with lattice parameters of *a* = 3.978 Å and *c* = 4.057 Å (*c*/*a* = 1.020).

**Figure 3.**
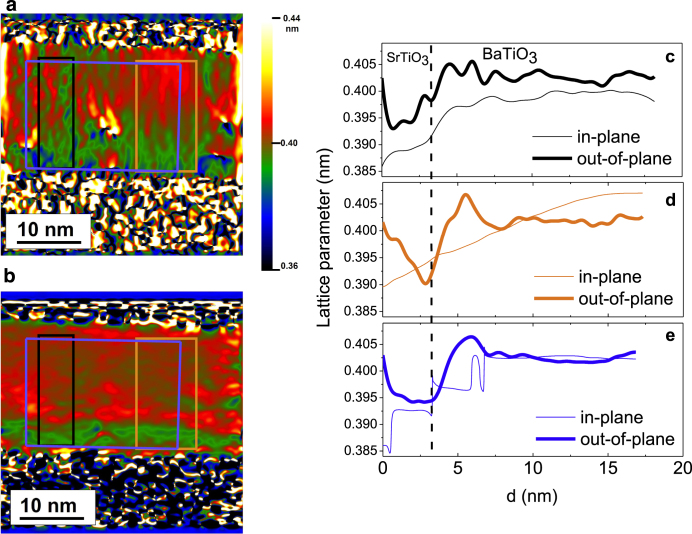
Strain analysis in a 16 nm BaTiO_3_/SrTiO_3_/amorphous interfacial layer (silicate and SiO_2_) stack. (a), (b) Maps of in-plane (a) and out-of-plane (b) lattice parameters determined from GPA of HR-STEM images. (c)–(e) Lattice parameter profiles as a function of distance *d* from the interface between the amorphous interfacial layer and the crystalline SrTiO_3_ layer, determined by averaging data from the black (c), orange (d) and blue areas (e) in (a) and (b). Adapted from figure 2 in [103]. Reprinted by permission from Macmillan Publishers: C Dubourdieu *et al* 2013 *Nat. Nanotechnol.* **8** 748, copyright 2013.

In [103], BaTiO_3_ films of thickness in the range 1.6–40 nm were studied with a 3.9–6.2 nm SrTiO_3_ template. X-ray diffraction and high-resolution TEM images indicated a pseudomorphic growth for the ultrathin 1.6 nm films. Films of thickness 8–10 nm were fully *c*-axis oriented with lattice parameters values close to the bulk ones (*a* = 3.993 Å and *c* = 4.038 Å with *c*/*a* = 1.011) while 16 and 40 nm films were composed of mixed *c*- and *a*-oriented domains. The local crystalline structure was determined by geometrical phase analysis (GPA) of high-resolution scanning transmission electron microscopy (HR-STEM) images. Figure 3 shows the lattice parameter maps along the [100] and [001] as well as the lattice parameter profiles as a function of distance from the amorphous interfacial layer determined for a 16 nm BaTiO_3_ film deposited on a 3.9 nm SrTiO_3_ buffer on Si. In figure 3(c), the out-of-plane lattice parameter is larger than the in-plane one throughout the BaTiO_3_ thickness, indicating fully *c*-axis oriented domain. In other regions of the film (figure 3(d) orange profile), the film grows first *c*-axis and switches to *a*-axis after ∼4.0–4.5 nm. It is noticeable that the in-plane parameter increases continuously and therefore the switch from *c*- to *a*-domains is continuous. The tetragonality is maximum close to the interface with the SrTiO_3_ template layer (e.g., *a* = 3.970 Å and *c* = 4.065 Å, *c*/*a* = 1.023) and decreases throughout the film thickness. Similar results were obtained on thicker films, in which the proportion of *a*-axis domains becomes predominant. Typical local lattice parameters determined by GPA were *a* = 4.01 Å and *c* = 4.05 Å (*c*/*a* = 1.010) for a 40 nm thick film. These values are similar to those reported in [101]. Edge dislocations were observed at the SrTiO_3_/BaTiO_3_ interface. The numerous profiles performed on different areas of each sample suggested that the lateral scale of the *c*- and *a*-domains, when in coexistence, was similar or smaller than the 10–20 nm lateral distance between dislocations.

In the work by Abel *et al* [104], a MBE 8 nm BaTiO_3_ film grown on 4 nm SrTiO_3_ was found to be fully *c*-axis oriented as well, with an out-of-plane lattice parameter close to the bulk *c* value. Similarly, in [105], we observed fully *c*-axis oriented films for 7 nm BaTiO_3_ with lattice parameters *a* = 3.996 Å and *c* = 4.027 Å. Droopad *et al* [106] reported *c*-axis orientation for a 8 nm film grown on a strained 2 ML (∼0.8 nm) SrTiO_3_ buffer, with an out-of-plane parameter *c* = 4.032 Å. Lattice parameters reported by various groups in this thickness range (7–8 nm) are in good agreement [103–106].

Thicker films of 80–130 nm were studied for photonics applications as they are of potential interest for integrated electro–optic modulators and other photonic devices [107, 108]. A 130 nm thick BaTiO_3_ film was grown on 4 nm thick SrTiO_3_ template and was fully *a*-axis relaxed with lattice parameters *a* = 3.997 ± 0.005 Å and *c* = 4.032 ± 0.005 Å (*c*/*a* = 1.009) [107]. For 80 nm BaTiO_3_ film on 8 nm SrTiO_3_-buffered silicon-on-insulator (SOI) substrates, an out-of-plane lattice constant of 3.998 Å and an average in-plane lattice constant of 4.03 Å were reported, indicating relaxed *a*-axis film as well [108]. Atomic force microscopy (AFM) showed a smooth surface with a root-mean square (rms) roughness as low as 0.4 nm (one unit cell) [108]. Such thick films are *a*-axis oriented due to the tensile in-plane biaxial strain applied by the substrate during cooling down.

**Figure 4.**
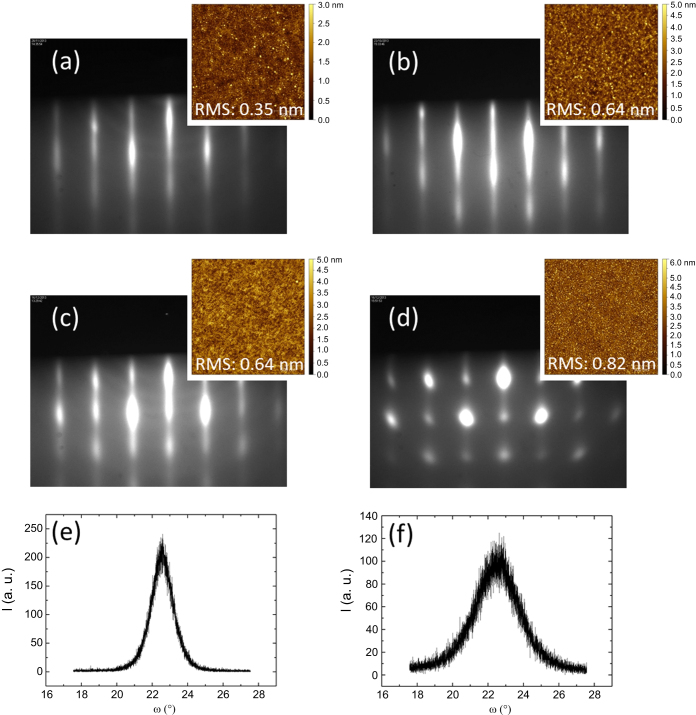
(a)–(d) RHEED patterns recorded along the [100] azimuth during BaTiO_3_ growth at 450 °C under an oxygen pressure of (a) 1 × 10^−7^ Torr, (b) 5 × 10^−7^ Torr, (c) 2 × 10^−6^ Torr, (d) 3 × 10^−6^ Torr and corresponding AFM images of the film surfaces. (e)–(f) Rocking curve measured on the 002 peak for the films grown at (e) 5 × 10^−7^ (FWHM = 1.5°) and (f) 2 × 10^−6^ Torr (FWHM = 2.9°).

The critical thickness at which the orientation switches from *c*- to *a*-axis is determined by the competing influence of compressive stress from epitaxy and tensile stress from thermal expansion. Among other crucial influence might be the SrTiO_3_ buffer thickness and surface quality and the composition of the BaTiO_3_ film. Slight cationic off-stoichiometry may result in oxygen vacancies and structural defects that impact the lattice parameters and strain state [32]. Deposition conditions such as oxygen background pressure or deposition temperature have a major influence on the film growth and may impact the film composition. We have shown [105] that oxygen partial pressure P(O_2_) has a strong effect on the morphology and crystalline orientation of 16–18 nm films. Increasing P(O_2_) in the range 1 × 10^−7^–3 × 10^−6^ Torr leads to an increase of the surface roughness as shown in figures 4(a)–(d) by the RHEED patterns and AFM images. The RHEED patterns for films grown at (1–5) × 10^−7^ Torr exhibit well-contrasted streaky lines as expected for a 2D growth. Starting at 2 × 10^−6^ Torr, partial or fully spotty patterns are recorded, which characterizes a rougher surface. From AFM, the rms increases from 0.35 nm (1 × 10^−7^ Torr) to 0.82 nm (3 × 10^−6^ Torr). X-ray diffraction indicated that increasing P(O_2_) promotes the growth of *a*-axis grains. Films grown at 1 × 10^−7^ Torr were fully *c*-axis oriented. With increasing P(O_2_), the out-of-plane parameter was found to decrease while the in-plane parameter increases. The FWHM of the rocking curves performed on the 002 peak (shown in figures 4(e)–(f)) is of 1.5° and 2.9° at 5 × 10^−7^ and 2 × 10^−6^ Torr respectively (our lowest FWHM for a 002 rocking curve measured for ∼16 nm films is of the order of 0.7°). The ratio of the out-of-plane/in-plane parameters is lower than 1 for pressures equal or larger than 2 × 10^−6^ Torr [105]. This trend was also reported for laser MBE-grown BaTiO_3_ films on SrTiO_3_ bulk substrates [109]. The effect of P(O_2_) on the cationic Ba/Ti composition and on its impact on the crystalline orientation should be further investigated.

**Figure 5.**
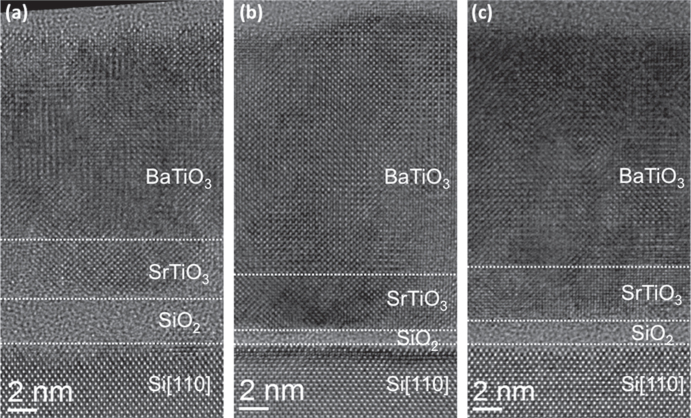
High resolution transmission electron microscopy images of BaTiO_3_/SrTiO_3_ stacks grown under P(O_2_) = 1 × 10^−7^ Torr for different temperatures and post-deposition process. (a) 450 °C—slow cooling down procedure at P(O_2_) = 1 × 10^−5^ Torr, (b) 440 °C—rapid cooling down under ultrahigh vacuum (UHV) followed by annealing under an oxygen plasma (1 × 10^−5^ Torr) for 40 min, (c) 525 °C—rapid cooling down under UHV followed by annealing under an oxygen plasma (1 × 10^−5^ Torr) for 40 min. A SiO_2_ interfacial layer between Si and SrTiO_3_ is formed upon SrTiO_3_ annealing and BaTiO_3_ growth and its thickness depends on the cooling down conditions. Horizontal dotted lines are only to guide the eyes. From figure 6 in [105]. Reprinted with permission from L Mazet *et al* 2014 *J. Appl. Phys.* **116** 214102. Copyright 2014, AIP Publishing LLC.

Oxygen stoichiometry is a major issue in MBE since oxidizing atmosphere and ultrahigh vacuum conditions are antagonistic. BaTiO_3_ is grown either using molecular O_2_ or atomic oxygen often created by a radio-frequency plasma. A post-deposition annealing might be performed in order to ensure sufficient oxidation of the films in order to reduce leakage currents and favor a stable ferroelectric polarization. Another issue related to the oxidation of the film is the SiO_2_ regrowth, which—depending on the application—might be detrimental for the properties. Thickness values of ∼3 nm [104] to ∼3.6 nm [103, 106] have been shown by TEM. Growth conditions and post-deposition annealing conditions have actually a strong impact on the interfacial SiO_2_ regrowth, as reported in [105] and illustrated in figure 5. We performed post-deposition annealing either in molecular O_2_ or in an oxygen plasma (typically 400 W). Figure 5(a) shows an interfacial layer of ∼2.5–3.0 nm for films grown at 450 °C under molecular O_2_ and slowly cooled down (10 °C min^−1^) to room temperature under P(O_2_) = 1 × 10^−5^ Torr. For the same growing temperature and P(O_2_) conditions during the growth but a different post annealing using a rapid cooling down under ultrahigh vacuum followed by a plasma anneal at 200 °C for 40 min, the SiO_2_ layer is only ∼0.7–1.0 nm (figure 5(b)). In the same post-deposition annealing conditions but at a growing temperature of 525 °C, the SiO_2_ is of ∼1.7 nm as indicated in figure 5(c). The use of an atomic oxygen plasma at low temperature clearly minimizes the interfacial layer regrowth [105]. A detailed study of the defect structure in the SrTiO_3_ buffer and BaTiO_3_ film is underway to determine the impact of the processing conditions.

**Figure 6.**
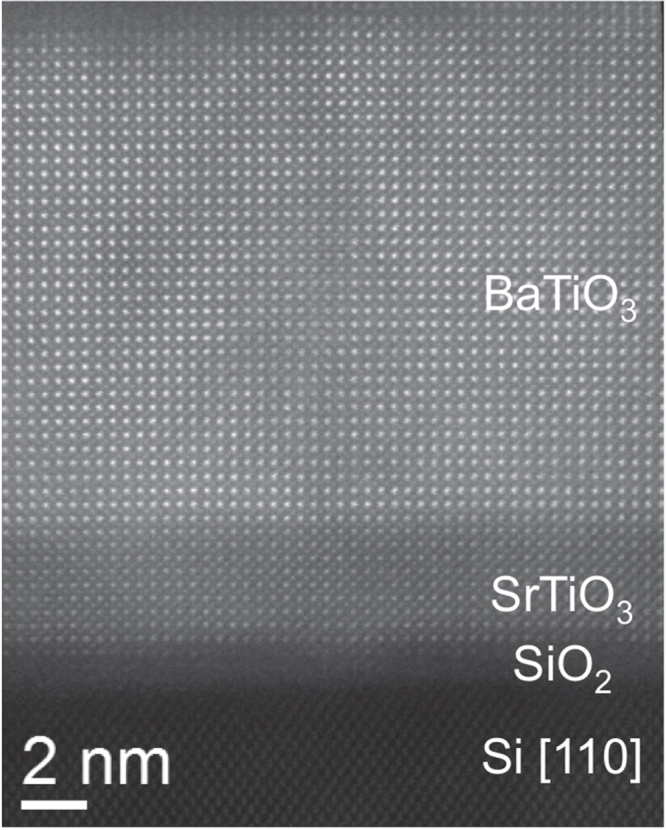
Scanning transmission electron microscopy high-angle annular dark field (HAADF) image of a BaTiO_3_/SrTiO_3_ stack grown on Si (001), indicating a sharp interface between SrTiO_3_ and BaTiO_3_ and a high crystalline quality of the perovskite oxides.

Figure 6 is a STEM high-angle annular dark field (HAADF) image of the sample shown in figure 5(b), illustrating the high crystalline quality of the perovskite stack and sharp BaTiO_3_/SrTiO_3_ and SiO_2_/SrTiO_3_ interfaces. The BaTiO_3_ film is coherently strained to the SrTiO_3_ buffer layer with no dislocations observed at the interface or in the film thickness.

In view of the literature data and various processing conditions used by the different groups, particularly regarding the oxidizing atmosphere (atomic or molecular oxygen and partial pressure), there is a need to better understand how to precisely control *c*- versus *a*-axis orientation in epitaxial films on Si as well as the defect chemistry, which further determine the ferroelectric properties of the films.

### 3.2. MBE of BaTiO_3_ on germanium

BaTiO_3_ exhibits a much lower mismatch with Ge (001) as compared to Si (001) (∼1.8% at room temperature), which allows the direct growth of high crystalline quality BaTiO_3_ without using a buffer layer. Moreover, Ge (001) is less prompt to oxidize than Si(001). However, in contrast to Si, the lattice mismatch with Ge leads to an in-plane tensile strain, which is not in favor to *c*-axis growth. In addition, the thermal expansion mismatch between BaTiO_3_ and Ge imparts an in-plane tensile stress to the film upon cooling down (Ge: *α* = 5.9 × 10^−6^ K^−1^).

McKee *et al* first demonstrated the epitaxial growth of BaTiO_3_ directly on Ge with a perfect pseudomorphic structure [110]. However, such films exhibited large leakage currents (of the order of ∼0.4 A cm^−2^ at −1 V for a 25 nm thick film). The insertion of 6 MLs of BaO at the interface between Ge and BaTiO_3_ led to a decrease of 6 orders of magnitude of the leakage currents [110]. From photoelectron spectroscopy, a valence band offset of 2.8 eV for BaTiO_3_ grown directly on Ge was reported. About 10 years later, further experimental works have been reported. The formation of alkaline-earth template layers on Ge(100) has been studied in detail in [111]. Both Ba and Sr have been used to promote the growth of BaTiO_3_ on Ge. Merckling *et al* grew BaTiO_3_ on Ge-on-Si (001) substrate (1 *μ*m thick fully relaxed epitaxial Ge layer on Si) using ½ ML Ba as a passivation layer [112]. In a 12 nm BaTiO_3_ film, they observed two different out-of-plane parameters of 4.072 and 4.060 Å and two in-plane parameters of 4.05 and 4.01 Å. They attributed these parameters to the presence of a tetragonal phase (*c*-axis oriented) with parameters close to the bulk one and to a cubic phase. Recently, Fredrickson *et al* [113] reported the growth of BaTiO_3_ on bulk Ge (001) substrates using a careful Ge surface preparation described in [114] and ½ ML Sr. The BaTiO_3_ films were deposited at 650 °C following three different stages (alternating the Ba and Ti fluxes) by progressively increasing the oxygen pressure from 1.5 × 10^−7^ to 5.10^−6^ Torr [113]. The films grown in these conditions were *a*-axis oriented. An out-of-plane parameter of 3.995 Å and an in-plane parameter of 4.01 Å were measured for a 40 nm BaTiO_3_ film (averaging the *a* and *c* values of the 90° in-plane domains) and the FWHM of the 200 rocking curve was ∼0.7°. From XPS measurements, the valence band offset between BaTiO_3_ and Ge was found to be 2.7 ± 0.1 eV, a value close to the one reported in [110]. Both in [110] and [113], high-resolution TEM images show an atomically sharp interface between Ge and BaTiO_3_.

In order to obtain BaTiO_3_ *c*-axis growth on Ge, it is necessary to insert a buffer layer at the interface that can impart a compressive in-plane strain. Ngai *et al* [115] have grown a 20 nm tri-layer Ba_1−*x*_Sr_*x*_TiO_3_ stack—with decreasing *x* values—as a buffer and have obtained *c*-axis oriented 40 nm thick BaTiO_3_ films. The in-plane and out-of-plane parameters were 3.987 Å and 4.040 Å respectively. Ponath *et al* [116] have grown *c*-axis BaTiO_3_ films using a 2 nm SrTiO_3_ buffer on Ge (with ½ ML Sr prior to the SrTiO_3_ buffer growth). The lattice parameters were *a* = 3.96 Å and *c* = 4.06 Å for 16 nm thick BaTiO_3_. Both in [115] and [116], the comparison of the x-ray diffraction *θ*/2*θ* scans clearly showed the impact of the buffer insertion on the BaTiO_3_ crystalline orientation. In the work of Ponath *et al* [116], STEM-HAADF images revealed that Ti atomic columns close to the top of the BaTiO_3_ film are shifted downward from the cell center, meaning a ‘down’ polarization, which is in good agreement with their DFT calculations. In contrast to this result and to the macroscopic mono-domain polarization of the as-deposited film, which is also shown to be oriented downward, the Ti atomic columns close to the SrTiO_3_ film are found to be shifted upward. From these images, it was also observed that no germanium oxide interfacial layer was formed at the interface between SrTiO_3_ and Ge [116]. The absence of low permittivity interfacial layer makes this structure particularly suited for negative capacitance devices as those described later in section 5.2.

As we have discussed the growth of BaTiO_3_ on Si and Ge, it is worth pointing out that SiGe alloy-based wafers should be of particular interest to engineer the strain and the interfacial layer in epitaxial BaTiO_3_.

### 3.3. MBE of BaTiO_3_ on gallium arsenide

Although the lattice mismatch between GaAs and BaTiO_3_ is similar to the one between Ge and BaTiO_3_, we are not aware of any report of direct epitaxial growth of BaTiO_3_ on GaAs by MBE. The epitaxial growth of BaTiO_3_ on GaAs has been performed via a buffer layer, in order to avoid interfacial reactions and to impart, like on Si and Ge, a compressive stress during cooling down in order to obtain *c*-axis oriented films (GaAs: *α* = 5.8 × 10^−6^ K^−1^).

Various oxide buffers have been studied to grow crystalline epitaxial oxides on GaAs (001). Laser MBE (base pressure 1 × 10^−7^ Torr) [117, 118] or MBE [119, 120] were used to grow MgO on GaAs for subsequent BaTiO_3_ growth by pulsed laser deposition. The following epitaxial relationship was obtained: MgO (001)//GaAs (001) and MgO [100]//GaAs [100]. MBE under molecular oxygen led to a reaction between Mg and GaAs and to a highly three-dimensional growth with a rough final surface morphology [119]. Following the growth of SrTiO_3_ on Si by MBE, routes have been also developed to grow high quality SrTiO_3_ films on GaAs.

#### 3.3.1. SrTiO_3_ epitaxial template on GaAs

SrTiO_3_ has been epitaxially grown by MBE on GaAs in the early 2000s using ½ ML Ti as a surface treatment (while ½ ML Sr is used on Si) [121, 122]. GaAs was first heated to about 600 °C in the presence of As_4_ flux to remove the native oxide layer. A homoepitaxial GaAs layer (∼0.5 *μ*m) was then grown. Prior to SrTiO_3_ deposition, ½ ML Ti was deposited at ∼300 °C. Both As- and Ga-terminated GaAs (001) surfaces were used [121]. Sr and Ti were co-deposited on the Ti-passivated GaAs surface in conditions that could preserve the surface: similarly to the conditions used for SrTiO_3_ deposition on Si, low-temperature (∼300 °C) and low 10^−8^ Torr O_2_ pressure. Both temperature and oxygen pressure were slowly ramped up as the deposition proceeded. Similarly to SrTiO_3_ deposition on Si, SrTiO_3_ was annealed after the first few MLs at ∼550 °C to be fully crystallized. Once these first steps were completed, the growth of SrTiO_3_ was then resumed at higher temperature. SrTiO_3_ grows on GaAs (001) with an in-plane 45° rotation of the cell as well. From high resolution TEM, the interface was found to be abrupt, free of interfacial Ga-oxide [121, 123]. The electronic structure of the interface was investigated by x-ray and ultraviolet photoelectron spectroscopy [122, 123]. The authors concluded that the Fermi level is pinned at the SrTiO_3_/GaAs interface when SrTiO_3_ is grown directly on GaAs while it is unpinned if ½ ML Ti is used prior to SrTiO_3_ deposition. However, band bending in GaAs was found to be very sensitive to the annealing conditions making integration of such materials challenging since the integrity of the interface could be strongly impacted by higher thermal budget steps required in device fabrication [122].

Other groups have reported the growth of SrTiO_3_ on GaAs [124–127]. Wu *et al* performed the growth by laser MBE without Ti pre-deposition, by ablating a SrTiO_3_ single crystalline target [124]. Louahadj *et al* [125, 126] performed the growth of SrTiO_3_ by MBE on *c*(4 × 4) As-terminated GaAs (001) surface using ½ ML Ti prior to SrTiO_3_ deposition. Such layers were then used as a template for subsequent La_0.7_Sr_0.3_MnO_3_/PZT stack deposition by pulsed laser deposition [126]. Contreras-Guerrero *et al* [127] studied the interface properties (Fermi level pinning) of films grown in different oxygen conditions on *c*(4 × 4) As-stabilized GaAs (001) surface with ½ ML Ti pre-deposition: first, 2 nm of SrTiO_3_ was grown under molecular oxygen and then the growth was continued either under molecular oxygen or under atomic oxygen. From room temperature photoluminescence experiments, they reported that the density of interfacial defects increased when an oxygen plasma was used and that the Fermi level was pinned similarly to that of a GaAs layer with a native oxide. *In situ* photoemission experiments showed an increase in the Ga–O bonding at the interface when atomic oxygen was employed as well as As–As bonding (not present under molecular oxygen). This study showed the crucial role of oxygen species during growth in determining not only the stoichiometry of the oxide but also the interface structural and electrical quality.

#### 3.3.2. MBE of BaTiO_3_ on SrTiO_3_-buffered GaAs

BaTiO_3_ has been deposited on SrTiO_3_-buffered GaAs substrates by MBE [106, 128] or by laser MBE [129, 130]. Huang *et al* reported the growth by laser MBE of *c*-axis oriented films [129]. They measured P–E loops using p-type GaAs as a bottom electrode and Pt as a top electrode. The loop exhibited a small concavity and was not saturated. The remanent polarization was 2.5 *μ*C cm^−2^ (with a maximum field of 600 kV applied during the measurement). Contreras-Guerrero *et al* reported the growth by MBE of BaTiO_3_ on n + GaAs substrates with a 2 unit-cell (8 Å) SrTiO_3_ buffer layer [128]. Ba and Ti were co-deposited under molecular oxygen at 500 °C under P(O_2_) of 1 × 10^−7^ mbar (7.5 × 10^−8^ Torr). Films of thickness 75 Å were *c*-axis oriented with an out-of-plane parameter of 4.032 Å.

Due to the interest in combining ferroelectrics with III–V compounds for optoelectronic applications, more work is to be expected in this area.

## 4. MBE of BaTiO_3_ on semiconductors: ferroelectricity

In ferroelectric thin films, charges induced by the polarization at the top and bottom interfaces may not be compensated or only partially compensated, which gives rise to a depolarization field. Boundary conditions are of utmost importance in determining the charge screening and depolarization field. It was shown [131] that the depolarization field arising in a ferroelectric thin film sandwiched between semiconducting electrodes significantly modifies the transition temperature, the spontaneous polarization amplitude and the coercive field. Under a critical film thickness, the switchable polar state becomes unstable [131].

While ferroelectricity of BaTiO_3_ on oxide substrates has been extensively studied, there are still few data for epitaxial films directly grown on semiconductors or on SrTiO_3_ (and other dielectric) buffered-semiconductors.

For a metal-oxide-semiconductor capacitor, a hysteresis of the capacitance versus voltage (*C*–*V*) curve is expected if the oxide is ferroelectric, with a clockwise and anti-clockwise hysteresis on respectively p- and n-type silicon [132]. In [102] *C*–*V* measurements were performed on a 40 nm BaTiO_3_ *c*-axis oriented film deposited on SrTiO_3_-buffered Si with 100 × 100 *μ*m^2^ top electrode area (with a final ∼2 nm SiO_2_ interfacial layer regrowth). No ferroelectric hysteresis was observed, which was attributed to the limited oxygen pressure during the MBE growth [102]. However, such measurements are usually not appropriate to evidence ferroelectricity when an interfacial layer such as SiO_2_ is formed. Indeed, the voltage applied across the heterostructure is mainly dropped in this low-permittivity (low *κ*) interfacial SiO_2_ layer (*κ* = 3.9 compared to *κ* > 250 for BaTiO_3_ films). The silicon also contributes to the total capacitance in depletion. Hence, it is not possible to reach an effective electrical field larger than the coercive field to switch the thin ferroelectric layer.

Piezoresponse force microscopy (PFM) has emerged as a major technique for the study of ferroelectricity at the nanoscale [133–135]. The feasibility of domain writing/reading, domain stability with time as well as the existence of piezoelectric hysteresis loop, checked by PFM, is an important necessary condition for ferroelectricity. However, it has been shown also that ionic and electrochemical phenomena may play a major role in scanning probe microscopy and can also lead to ferroelectric-like domain writing/reading and hysteresis loop [136, 137]. For example, those features were observed in non-ferroelectric compounds such as crystalline LaAlO_3_/SrTiO_3_ heterostructures [138] or transition metal oxides involved in memristive devices like TiO_2_ or SrTiO_3_ [139]. In case of conventional ferroelectrics such as BaTiO_3_ (well known in bulk), complementary structural information in thin films are useful. The dependence of the PFM signal with input voltage and film thickness should also be checked.

Several groups have reported evidences by PFM consistent with ferroelectric switching for MBE-grown BaTiO_3_ on SrTiO_3_-buffered-Si [71, 103–106], -Ge [116] and -GaAs [106, 128] substrates.

**Figure 7.**
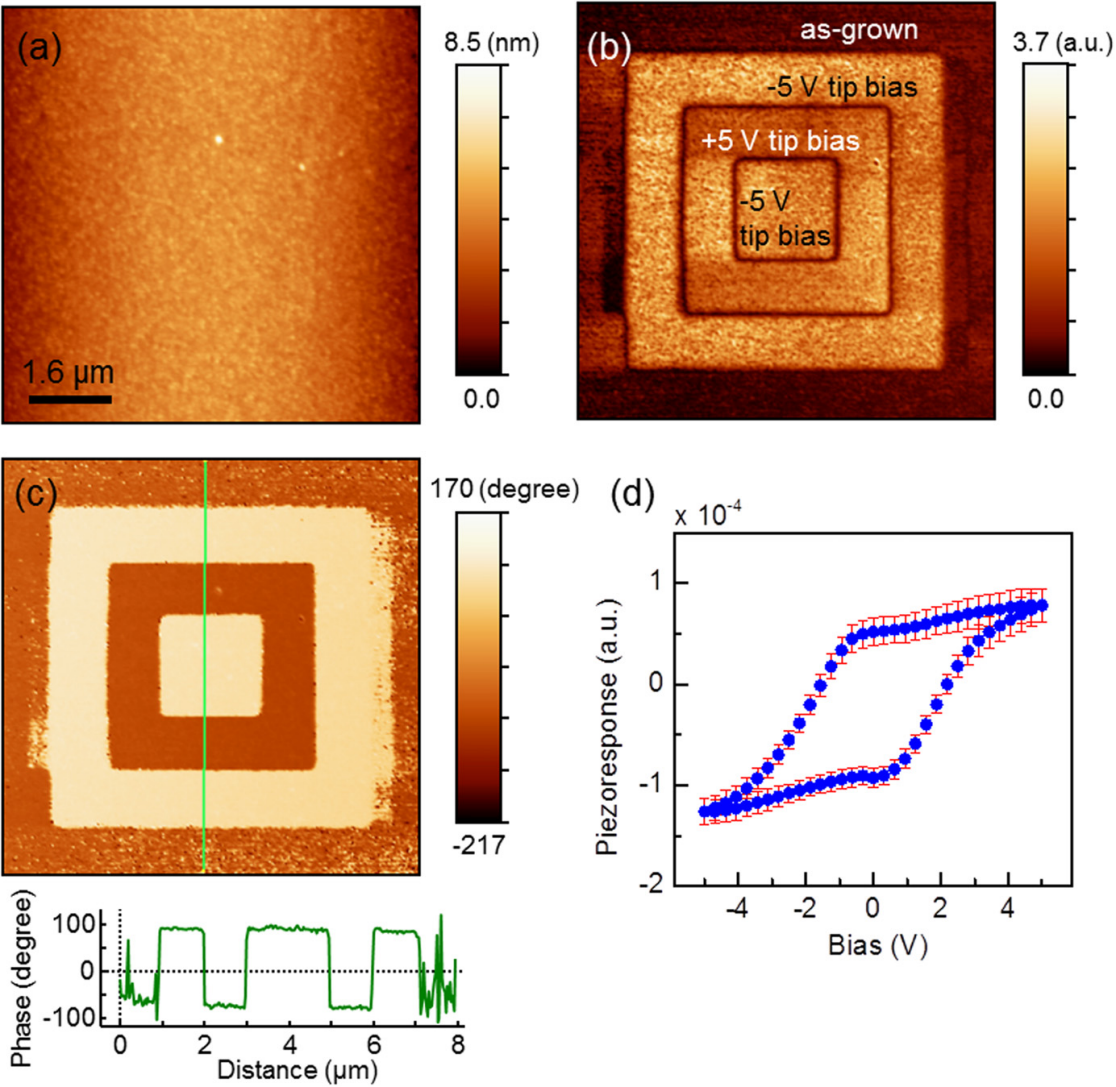
(a) Atomic force microscopy topography and piezoresponse force microscopy (b) amplitude and (c) phase images for 17 nm thick BaTiO_3_/SrTiO_3_ (∼4 nm) on Si substrate, poled with −5 V, +5 V, and −5 V over 6 *μ*m, 4 *μ*m, and 2 *μ*m regions, respectively. The images were collected over 8 × 8 *μ*m^2^ areas. The bottom panel in (c) shows the line profile of phase signals, exhibiting clear phase difference of ∼180 degrees. (d) Piezoresponse hysteresis loop averaged over 10 × 10 points within 4 × 4 *μ*m^2^ area. Error bars represent the dispersion of the signal measured at different locations on the sample surface.

Figures 7(b)–(c) show typical PFM images (amplitude and phase respectively) for a 17 nm thick *c*-axis BaTiO_3_ film grown on SrTiO_3_ (∼4 nm) on Si substrate, poled with −5 V, +5 V, and −5 V over 6 *μ*m, 4 *μ*m, and 2 *μ*m regions, respectively. Figure 7(b) indicates similar amplitudes for +P and −P signals with a zero signal at the boundary between opposite poled regions and the graph of figure 7(c) shows a clear phase difference of ∼180° between +P and −P regions. The as-deposited film (non-poled regions) does not appear mono-domain. A piezoresponse hysteresis loops consistent with ferroelectricity is shown in figure 7(d). The coercive voltages are of ∼ −1.8 V and +2 V.

For BaTiO_3_ grown directly on Ge substrates, no ferroelectricity was reported from electrical or electromechanical measurements with an applied electric field perpendicular to the film, which can be related to the fact that the growth is *a*-axis oriented [110, 116]. In the work by Merckling *et al* [112] where a mixture of both *c*-axis tetragonal phase and cubic phase was reported, ferroelectricity was not studied. Ferroelectricity was reported for BaTiO_3_ *c*-axis oriented films on (Ba,Sr)TiO_3_-buffered Ge [115, 116]. Current versus voltage curves were measured on capacitive structures with 40 nm BaTiO_3_ deposited on a trilayer Ba_1−*x*_Sr_*x*_TiO_3_ buffer (20 nm) stack and with top Pt electrodes, showing rectifying behavior [115]. A hysteresis consistent with ferroelectric switching was observed while no hysteresis was present for a heterojunction with *a*-axis 60 nm BaTiO_3_ directly on Ge [115]. In *c*-axis 16 nm BaTiO_3_ film on 2 nm SrTiO_3_ buffered-Ge [116], PFM measurements indicated ferroelectricity with a coercive voltage of −4 V and +5 V. The non-poled regions (as-deposited film) were found to be mono-domain with the polarization oriented towards the STO/Ge substrate, which was in agreement with their theoretical calculations [116]. In addition to the ferroelectricity of BaTiO_3_, they also demonstrated the ferroelectric field-effect on the conductivity of the underlying Ge using microwave impedance microscopy [116].

Similarly, ferroelectricity in BaTiO_3_ *c*-axis film (7.5 nm) on 0.8 nm SrTiO_3_-GaAs substrate was inferred from PFM measurements, with a repeatedly switchable polarization [128]. Patterns written and read were stable over ∼1 h. The coercive voltage was of the order of ±1–2 V. As-deposited films were poled with a polarization pointing towards the bottom interface [128].

Regarding the dependence of ferroelectricity on the thickness of ultrathin films, very limited work is reported. The thickness dependence in the range 1.6–40 nm of the PFM hysteresis loops was studied in [103] for BaTiO_3_ on SrTiO_3_-Si. Films of thickness 40 nm showed closed and saturated hysteresis loops. As the thickness was decreased down to 8 nm, the hysteresis loops were still well-defined with an elongated shape consistent with depolarization field effect [140]. An offset of the electromechanical signal was also observed as thickness decreased [103], which was attributed to imprint phenomenon originating from regions with non- or non-fully-switchable polarization. The coercive voltages for the 16 nm film were of the order of −10 V and +6 V [103], asymmetric and much larger than the ones obtained in the measurement shown in figure 7 for a similar thickness (and for a same SrTiO_3_ buffer layer thickness of 4 nm). This may be due to the mixed *c-* and *a-*domain structure in the study reported in [103] while the film shown in figure 7 is fully *c*-axis oriented. Fully *c*-axis oriented films of 8 nm (with a SrTiO_3_ buffer of 6.2 nm) had coercive voltages of ∼ ±4 V [103].

The ferroelectric polarization of thin and ultrathin films, the coercive field and the ferroelectric domain configuration are strongly dependent on the film thickness as well as on the boundary electrical conditions (nature of the electrodes) and polarization charge screening [141–146]. Garcia *et al* showed that an ultrathin 1 nm BaTiO_3_ film epitaxially grown on a metallic manganite electrode is ferroelectric [145]. First-principle computations show that a net positive polarization exists in ultrathin SrTiO_3_, BaTiO_3_ or PbTiO_3_ epitaxial films on silicon but that it cannot be switched as it is pinned by the interface [147]. We recently observed ferroelectricity in ultrathin BaTiO_3_ films down to 1.6 nm on SrTiO_3_-buffered silicon [148] but with a strong imprint. For a same film thickness, the thickness of the SrTiO_3_ buffer layer and the defect chemistry in such layer certainly play a major role in the polarization stability and amplitude in the BaTiO_3_ film, which is currently under investigation.

Compared to the studies performed on BaTiO_3_ films grown on oxide substrates (such as SrTiO_3_ or NdGaO_3_ bulk crystals), a major difference on growing on semiconductors resides in the strong tensile strain imparted to the film during the cooling time, which may strongly affect the strain state, the defects, the crystalline phase(s) and the crystalline orientation(s) stabilized, which, in turn, impact the ferroelectric properties. Ferroelectric domains in thin BaTiO_3_ films on silicon and other semiconductors need to be further explored in order to control their size and distribution.

## 5. Application of BaTiO_3_ epitaxial films on semiconductors

There are many applications for which ferroelectric epitaxial films on semiconductors can bring new functionalities. As a piezoelectric material, it can be integrated into microelectromechanical systems (MEMS) to design actuators, transducers or sensors [149]. Non-volatile memories have been one of the major application areas of ferroelectrics [150]. Integration of a ferroelectric on silicon offers the ability to fabricate ferroelectric-FETs (FeFETs) as memory or logic devices. We will focus here on two recent areas that have generated several works on the monolithic integration of BaTiO_3_ on semiconductors. One growing field of interest is in integrated photonics on silicon where building blocks such as electro–optic modulators could benefit from the high Pockels effect of BaTiO_3_. The other one is in the realization of low power logic devices that has been suggested using the negative capacitance of ferroelectrics.

### 5.1. Integrated photonics applications

Ferroelectrics are highly attractive for integrated optics to design waveguides with low losses and high bandwidth electro–optic modulators due to their large electro–optic coefficients, optical transparency and thermal stability [151]. In an electro–optic modulator, the phase of the light travelling through the crystal changes depending on the applied electric field. In bulk, lithium niobate LiNbO_3_ is widely used as an electro–optic medium. Waveguides are designed by modifying the composition of the substrate through diffusion or ion exchange [152] with resulting devices of millimeter or centimeter size. Integrating optical communication functionalities using thin films, especially on silicon platform, stimulates considerable research efforts. Indeed, integration of epitaxial films on silicon offers the ability to co-integrate optical functionalities with standard CMOS ones. Hybrid silicon/lithium niobate optical microring resonators have been recently demonstrated [153–156]. However, the devices on silicon were fabricated from LiNbO_3_ bonded to silicon using complex techniques. Integrating epitaxial films in a monolithic route would offer much more flexibility. BaTiO_3_ is particularly attractive for such purpose. It presents high refractive indices (*n*_o_ = 2.412 and *n*_e_ = 2.36 at 633 nm) with superior linear electro–optic properties compared to LiNbO_3_, exhibiting one of the highest reported Pockels effect (*r*_113_ = 14.5 pm V^–1^, *r*_33_ = 103 pm V^–1^ and *r*_42_ = 1700 pm V^–1^ at *λ* = 633 nm from [157]—similar values are also reported in [158]).

Several studies have been conducted on BaTiO_3_ epitaxially grown on mainly MgO substrates (of lower optical index to allow optical confinement) to design waveguides and electro–optic modulators, either in ridge or strip-loaded configurations and have shown the potential of this material [159, 160]. Photonic crystal waveguide structures have been proposed to improve the performance of these devices [161–163]. In a recent work, Li *et al* showed potential for achieving modulation at 65 GHz [164]. The epitaxial growth of BaTiO_3_ on SrTiO_3_ buffered-silicon offers a great potential for performing integrated planar waveguides and electro–optic modulators as well as optical/ferroelectric combined functionalities. Abel *et al* reported recently for the first time the electro–optical properties of epitaxial BaTiO_3_ films on SrTiO_3_/silicon [107]. They showed that BaTiO_3_ exhibit a much higher effective Pockels coefficient of *r*_eff_ = 148 pm V^–1^ (*λ* = 1.55 *μ*m), at least five times larger than the one of LiNbO_3_. Recently as well, the first monolithically integrated BaTiO_3_ modulators on SOI substrates were reported [108, 165]. Since the silicon has a higher refractive index than BaTiO_3_, conventional ridge or strip waveguide configurations are not suitable. The design is therefore that of a horizontal slot waveguide in which the *a*-axis oriented BaTiO_3_ layer (80 nm) is comprised between the silicon substrate (110 nm Si from the SOI wafer) and an amorphous silicon layer (110 nm) [108]. The waveguide is patterned into the amorphous silicon layer and electrodes are patterned on each side of the waveguide. Mach-Zehnder interferometers and microring resonators were demonstrated [108]. The authors reported an effective Pockels coefficient of *r*_eff_ = 213 ± 49 pm V^–1^. Similar works pursuing the integration of BaTiO_3_ on silicon for electro–optic modulators are ongoing [166].

### 5.2. Low power logic device applications

Power dissipation is one of the major issues that the CMOS nanoelectronic industry is currently facing. For decades, transistor dimensions have been scaled down at constant electric field following Dennard’s rules [167]. Such scaling implied that the supply voltage be reduced and as a consequence, that the threshold voltage *V*_th_ of the transistor be reduced, leading after ∼2005 to unacceptable *I*_OFF_ leakage currents. In order to maintain a high enough *I*_ON_/*I*_OFF_ ratio (while the subthreshold swing is thermodynamically limited to 60 mV/dec at room temperature), the scaling rules have therefore been changed to maintain a constant supply voltage. New materials (high-k oxides, III/V semiconductors) and architectures (fully-depleted SOI technology, multiple gate FETs…) have so far allowed us to keep miniaturization compatible with performance although clock frequency has to be limited. The impossibility to further reduce the operating voltage leads to a more general societal issue of energy consumption in a world where individual consumers now posses several electronic products. The percentage of energy consumption by individuals compared to industry keeps growing. There is an urgent need for low-power logic switches that could operate at ∼0.2 V or below and several device concepts have been proposed [168].

In 2008, Salahuddin and Datta suggested that the negative capacitance of a ferroelectric could be used to decrease the subthreshold swing below 60 mV/dec [169]. Although the state of negative capacitance of a ferroelectric is unstable, it could be, however, possible to stabilize it by having in series a suitable positive capacitance. If the ferroelectric is inserted as a gate oxide in a FET and if its thickness is tuned to match the positive one of the silicon/dielectric (interfacial layer e.g.), the two contributions would cancel, leading to a very high effective capacitance. A small change in gate bias could therefore control a large change in the channel charge, meaning low voltage operation [169, 170]. There have been many experimental and theoretical works since this initial proposal [171–183]. A sub-60 mV/dec subthreshold swing has been demonstrated in a FET using a ferroelectric polymer [171, 177]. A thin AlSi metal was inserted between the ferroelectric layer and the SiO_2_ interfacial layer, acting as an internal electrode. Slopes ranging from 46 to 58 mV/dec were reported [171]. Several works have focused on combining ferroelectric and paraelectric epitaxial complex oxides in metal–insulator–metal (MIM)-type capacitive structures and demonstrated capacitance enhancement as compared to individual contributions, concluding to negative capacitance effect. Khan *et al* [174] reported an enhanced capacitance in a bilayer of Pb(Zr_0.2_Ti_0.8_)O_3_/SrTiO_3_ epitaxially grown on a conducting SrRuO_3_ electrode at a temperature larger than 500 K. In 2014, two groups have reported room-temperature capacitance enhancement in BaTiO_3_-based epitaxial heterostructures on SrRuO_3_. Appleby *et al* [179] studied BaTiO_3_/SrTiO_3_ bilayers and Gao *et al* [180] studied (LaAlO_3_/Ba_0.2_Sr_0.8_TiO_3_) superlattices. Recently, Khan *et al* [181] showed, for the first time, a direct proof for the negative capacitance in an epitaxial Pb(Zr_0.2_Ti_0.8_)O_3_ film on a metallic SrRuO_3_-buffered SrTiO_3_ substrate with a top Au electrode. The capacitive structure was put in series with a large resistance in order to be able to measure the transient region when the ferroelectric passes through the unstable negative capacitance state. As a voltage pulse was applied—while a regular capacitor would exhibit an increased voltage—the voltage across the capacitor was shown to decrease, thus indicating a negative capacitance transient [181].

**Figure 8.**
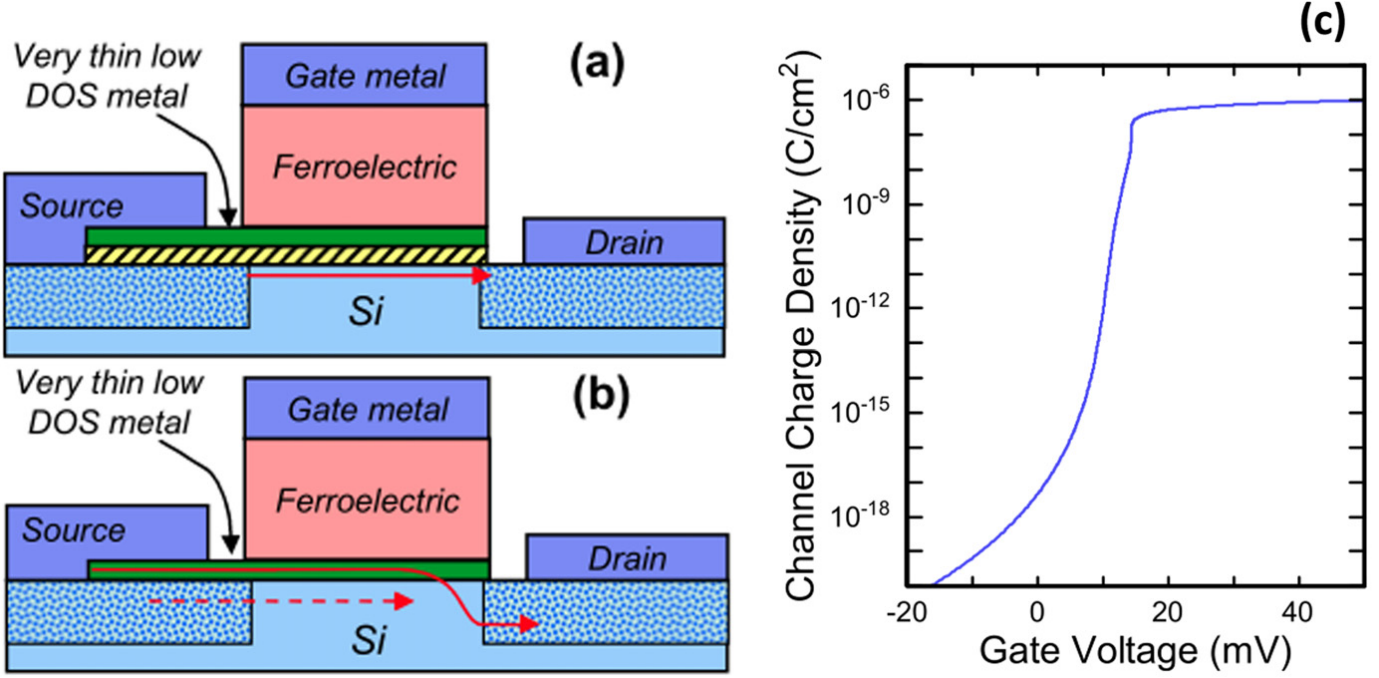
Device structures for FeFET with quantum metal layer. The arrows indicate current flow paths. (a) With and (b) without a thin insulator between the quantum metal and the semiconductor—(c) calculated *Q*–*V* curve for an FeFET as represented in (a)—DOS stands for ‘density of state’. Adapted from figures 4 and 6(a) in [178]. Reprinted with permission of IEEE (D Frank *et al* 2014 *IEEE Trans. Electron Devices* **61** 2145).

These demonstrations of negative capacitance in MIM structures based on complex ferroelectric oxides give insight into the materials and show that the concept of negative capacitance may hold promise for FET devices. There is, however, no realization so far of a transistor fabricated with a ferroelectric epitaxial oxide on silicon. The reason is the major difficulty to integrate a complex oxide in a transistor following a conventional gate first route. One major issue is the integrity of the ferroelectric after the high-temperature anneal that is required to activate the source and drain regions (typically 1065 °C in the current technologies). A replacement gate route, which experiences a much lower thermal budget, should be followed to save the oxide properties. Another issue is the SiO_2_ interfacial layer that is formed during BaTiO_3_ growth on SrTiO_3_-Si substrates. When as little as few Angström of the low permittivity SiO_2_ dielectric is formed, it requires the BaTiO_3_ layer thickness to be increased to few hundreds or few thousands of Angström to reach the capacitance balance. Finally, the concept of negative capacitance FeFET (NC-FeFET) has limitations, which are discussed in details in [178]. One major issue is that the capacitance of the silicon is strongly varying when going from depletion to inversion regimes while the capacitance of the ferroelectric is almost constant in the same voltage range, making the match impossible in the whole operating range. Another concern with using a ferroelectric complex oxide on silicon for NC purpose concerns the charge mismatch: when operating at low voltage, the charge change in Si between OFF and ON states is estimated to ∼0.2 *μ*C cm^−2^ while the ferroelectric switches much more charges (typically 2–20 *μ*C cm^−2^) as calculated in [178]. To address these issues, new devices concepts named ‘Quantum metal Fe-FET’ were proposed [178] and are shown in figure 8. A thin metal layer (called quantum metal) is inserted between the ferroelectric and the semiconductor and is intended to present a constant capacitance to the ferroelectric. It is designed such that its electron carrier density is low and can be modulated by the change in polarization of the ferroelectric layer. For a 2 nm metal layer, the carrier density should be of the order of 10^21^ cm^−3^, which could be achieved using doped SrTiO_3_ [45] or TaN_*x*_ films [184]. Two different devices were proposed [178]. In one case (device shown in figure 8(a)), the current flows in the semiconductor inversion layer like in a conventional FET, with the quantum metal’s modulated work function serving as the gate for the semiconductor. In the case of the device in figure 8(b), the current flows from the quantum metal into the silicon as in a Schottky barrier diode, with the barrier height being modulated by the FE gate electrode. A very steep slope in the channel charge versus gate voltage could be achieved, as shown in figure 8(c) from the modeling of the device shown in figure 8(a). The charge changes by 11 orders of magnitude (700 mV change in surface potential) for a 20 mV change in the gate voltage, for a slope of more than 500 decades V^–1^, or 2 mV/dec for the inverse slope. Hence, such devices are particularly attractive for future low power switches. However, technological challenges to fabricate such devices remain to be addressed.

## 6. Conclusions

We have reviewed studies on epitaxial BaTiO_3_ grown by MBE on Si, Ge and GaAs semiconducting substrates. The SrTiO_3_ buffer layer epitaxially grown on these substrates plays a key role to maintain a compressive strain to favor *c*-axis growth. High crystalline quality and ferroelectric properties were demonstrated on the three substrates. Advancing ferroelectric applications requires better control and understanding of the effect of oxygen and cationic composition on the ferroelectric properties. The domain pattern should be also further investigated in order to control their size and distribution. Engineering of bottom and top interfaces with the ferroelectric layer could offer possible paths to such control. Relatively few works have been done in growing a top metallic electrode *in situ* in order to possibly control the domain structure. Wetting of Pt on BaTiO_3_ (as a potential top electrode) has been studied by DFT and experimentally [185]. DFT showed that despite a reasonable match of the lattice constant, the surface energy of both (100) and (110) Pt is too high to wet BaTiO_3_, which was confirmed by TEM observations, showing Volmer–Weber faceted islands, epitaxial with the underlying BaTiO_3_ films [185]. Other metals such as TiN or TaN widely used in nanoelectronics should be investigated.

Regarding device fabrication, thick BaTiO_3_ films show promise in integrated photonics while thin films are of interest for low power logic devices. Heterostructures on Ge, in which no low-permittivity interfacial layer is formed, could be of particular interest to fabricate field-effect transistors with a steep subthreshold swing if the negative capacitance of the ferroelectric could be balanced with the one of the SrTiO_3_ and Ge contributions. Moreover, the ability to tune the SrTiO_3_ template to a conducting film using La^3+^ doping could be used for the design of the quantum metal field-effect transistor.

Progress in the epitaxial growth of perovskite compounds on semiconductors will also open up the route towards more complex heterostructures combining oxide and multiple semiconducting layers. Inserting a ferroelectric or piezoelectric oxide film in a semiconducting quantum well e.g. could enable to modify the electronic and optical properties of the well using ferroelectric field-effect or using piezoelectric strain. It was shown that the properties of a two dimensional electron gas can be modified e.g. by poling of a Cd_0.96_Zn_0.04_Te ferroelectric gate deposited on the top of a CdTe-based quantum well structure [186, 187] or with a LiNbO_3_ film on nitride heterostructures [188].

Not addressed here are ferroelectric or piezoelectric/piezotronic nanowires or nanopillars, which are of interest for energy harvesting and sensors applications [189, 190]. Other perspectives concern the use of domain walls in the ferroelectric epitaxial films on semiconductors to design specific devices based on new functionalities, not present in the domains [144]. Certain types of domain walls can be conducting while the domains are insulating and the domain walls can be controlled by an electric field [144, 191–195]. While works are progressing on ferroelectric/multiferroic perovskites grown on oxide substrates, nothing has been reported, to our knowledge, on silicon. Nanoelectronic based on domain walls would first require the ability to synthesize periodic arrays of domain walls with tunable densities on semiconductors. This area promises exciting future developments.
